# Acute Autonomic and Perceptual Responses to Resistance Training Performed With and Without Blood Flow Restriction

**DOI:** 10.3390/jfmk11020235

**Published:** 2026-06-12

**Authors:** Paulo H. da Silva Steiger, Tiago A. F. Almeida, Danilo A. Massini, Gabriel de Souza Zanini, David Michel de Oliveira, Víctor Hernández-Beltrán, José M. Gamonales, Mário C. Espada, Dalton M. Pessôa Filho, Anderson Geremias Macedo

**Affiliations:** 1 Postgraduate Program in Rehabilitation Sciences, Institute of Motricity Sciences, Federal University of Alfenas, Santa Clara Campus, Alfenas 37130-001, MG, Brazil; paulosteiger2018@gmail.com (P.H.d.S.S.); andersongmacedo@yahoo.com.br (A.G.M.); 2 Centre for Research in Economics and Comparative Development (CIEDEC), Lusíada University of Lisbon, 1349-001 Lisbon, Portugal; tiagofalmeida.w@gmail.com; 3 Department of Physical Education, School of Sciences, São Paulo State University (UNESP), Bauru, São Paulo 17033-360, SP, Brazil; dmassini@hotmail.com (D.A.M.); gabriel.zanini@unesp.br (G.d.S.Z.); 4 Department of Physical Education, Federal University Jataí (UFJ), Jataí 75801-615, GO, Brazil; profdoliveira@ufj.edu.br; 5 Postgraduate Program in Animal Bioscience (PPGBA), Institute of Health Sciences (ICS), Federal University of Jataí (UFJ), Jataí 75801-615, GO, Brazil; 6 Training Optimization and Sports Performance Research Group (GOERD), Faculty of Sport Science, University of Extremadura, 10005 Cáceres, Spain; victorhb@unex.es (V.H.-B.); martingamonales@unex.es (J.M.G.); 7 Faculty of Education and Psychology, University of Extremadura, 06006 Badajoz, Spain; 8 Instituto Universitario de Investigación e Innovación en el Deporte (INIDE), University of Extremadura, 10003 Cáceres, Spain; 9 Escola Superior de Educação, Instituto Politécnico de Setúbal (CIEQV Setúbal), 2914-504 Setúbal, Portugal; mario.espada@ese.ips.pt; 10 Sport Physical Activity and Health Research & Innovation Center (SPRINT), Sport Sciences School of Rio Maior (ESDRM), Instituto Politécnico Santarém, 2040-413 Rio Maior, Portugal; 11 Faculdade de Motricidade Humana, Centro Interdisciplinar de Performance Humana (CIPER), Universidade de Lisboa, 1499-002 Lisbon, Portugal; 12 Comprehensive Health Research Centre (CHRC), Universidade de Évora, 7000-645 Évora, Portugal; 13 Graduate Program in Human Development and Technology, São Paulo State University (UNESP), Rio Claro 13506-900, SP, Brazil

**Keywords:** resistance exercise, heart rate variability, rehabilitation, Kaatsu training

## Abstract

**Objectives:** This study aimed to compare the acute effects of high-intensity resistance training (HIRT), low-intensity resistance training (LIRT), and low-intensity resistance training with blood flow restriction (LIRT-BFR) on heart rate variability (HRV), rating of perceived exertion (RPE), total load (kg), and number of repetitions in young trained men. **Methods:** Thirteen volunteers (21.5 ± 1.6 years; 178.2 ± 8.0 cm; 75.7 ± 8.0 kg) performed three training sessions with six upper- and lower-limb exercises in repetition-to-failure mode. HIRT was performed at 70% 1RM, four sets and 90 s of rest; LIRT at 30% 1RM, four sets and 30 s of rest; and LIRT-BFR at 30% 1RM, four sets, 30 s of rest, and cuff pressure at 80 mmHg. The rest interval between training sessions was 72 h. **Results:** Total load was higher during LIRT compared with LIRT-BFR (*p* < 0.05), with no significant difference compared with HIRT (*p* > 0.05). The number of repetitions was greater in LIRT than in HIRT (*p* < 0.05), with no significant difference compared with LIRT-BFR (*p* > 0.05). RPE was lower in LIRT compared with HIRT and LIRT-BFR (*p* < 0.05). Time-domain parameters SDNN significantly decreased across all protocols (*p* < 0.001), whereas RMSSD showed no differences. Frequency-domain components (LFnu, HFnu, and LF/HF) showed no significant differences. **Conclusions:** LIRT elicited lower perceived exertion compared with HIRT and LIRT-BFR and higher repetition performance, whereas LIRT-BFR, despite showing similar autonomic responses, produced greater perceptual stress, resembling that of HIRT.

## 1. Introduction

High-intensity resistance training (HIRT), usually at 60% of one-repetition maximum [1RM], has been widely recommended for healthy adults to increase muscle strength and mass, promote overall health, and prevent chronic conditions [1,2]. However, a resistance training session is not defined only by relative load. The number of exercises, number of sets, inter-set recovery, exercise order, muscle groups involved, and whether sets are performed until concentric failure all contribute to the mechanical, metabolic, cardiovascular, and perceptual demands imposed by the session [3,4,5]. Current guidelines emphasize that HIRT sessions should include exercises targeting different body segments and muscle groups [1,2]. During HIRT, significant alterations occur across multiple physiological systems, particularly within the cardiovascular system. These alterations involve shifts in autonomic balance, with reductions in parasympathetic tone and increases in sympathetic activity to sustain the metabolic demands of the active muscles [6].

Several strategies have been used to monitor internal load and recovery during exercise, including heart rate-derived indices, heart rate reserve, training impulse models, rating of perceived exertion (RPE), heart rate variability (HRV), and more recently, composite approaches such as the WINT index. These tools do not provide interchangeable information [7,8,9,10,11,12,13]. For instance, heart rate reserve (HRR) is widely applied to prescribe and monitor cardiorespiratory intensity, showing a strong correlation with oxygen consumption reserve percentages [12], while composite approaches like the WINT index have recently emerged to integrate intensity and duration, particularly during high-intensity interval endurance training [10]. However, because these metabolic and heart rate-dependent models are predominantly tailored for continuous, long-duration cardiorespiratory exercise, their applicability to resistance training is limited. In contrast, rating of perceived exertion (RPE) and heart rate variability (HRV) are highly effective for monitoring intermittent, high-intensity strength protocols. While RPE reflects an integrated perceptual response influenced by central motor command, localized muscle fatigue, and psychological tolerance [7,9,12,13], HRV serves as a sensitive, non-invasive marker of cardiac autonomic modulation and systemic recovery [14,15,16], making them the most appropriate metrics for monitoring acute load of resistance exercise on autonomic and sensorial systems. In resistance exercise, RPE and HRV responses may be influenced by exercise intensity, volume, rest interval duration, muscle mass involved, breathing pattern, and proximity to concentric failure [13,17].

Low-intensity resistance training combined with blood flow restriction (LIRT-BFR) has emerged as an alternative strategy to increase metabolic stress while using relatively low external loads [18,19,20]. In this method, external cuffs are positioned proximally on the limb to partially restrict arterial inflow and reduce venous outflow during exercise [10,18]. This produces a local environment characterized by reduced oxygen availability, metabolite accumulation, and increased intramuscular pressure. These conditions may accelerate peripheral fatigue and increase activation of group III and IV muscle afferents, which are involved in the exercise pressor reflex and contribute to cardiovascular and perceived exertion during exercise [21,22,23,24,25].

The LIRT-BFR has received considerable attention because it can induce meaningful neuromuscular adaptations despite the use of very low external loads (~20–30% 1RM) [19,20,21,22]. Acutely, LIRT-BFR is characterized by substantial metabolic stress, metabolite accumulation, local hypoxia, and elevated motor-unit recruitment despite the low mechanical load [22,24]. These physiological characteristics may result in perceptual responses similar to, or in some cases greater than, those observed during traditional high-intensity resistance exercise [23], while cardiovascular responses may be maintained or attenuated [26,27]. Previous evidence indicates that training with LIRT-BFR can promote significant increases in muscle strength and hypertrophy [28], with hypertrophic adaptations approaching those observed with traditional high-intensity resistance training during short-term interventions (≤8 weeks), typically in the range of 6–7% increases in muscle size [29,30]. Importantly, the application of LIRT-BFR extends beyond healthy young adults and has been investigated in older adults, clinical populations, musculoskeletal rehabilitation settings, and athletes during injury recovery or periods of reduced load tolerance [24,25]. Because it allows the maintenance of a robust internal stimulus while minimizing external mechanical stress, LIRT-BFR represents a valuable alternative to traditional resistance training when high loads are contraindicated or poorly tolerated [25,28]. Therefore, although both LIRT-BFR and high-intensity resistance training may promote favorable neuromuscular adaptations [29], they do so through distinct mechanical, metabolic, perceptual, and cardiovascular pathway [22,27].

This is particularly relevant for individuals who cannot tolerate high joint or musculoskeletal stress, such as patients in rehabilitation, older adults, or individuals returning to training after injury [17,24,25]. Nevertheless, the same physiological mechanisms that make BFR effective may also increase acute discomfort, perceived exertion, and cardiovascular/autonomic strain [22,26]. Therefore, evaluating both perceptual and autonomic responses is important to determine whether LIRT-BFR represents a tolerable and safe alternative to traditional resistance training strategies.

However, studies comparing HRV and RPE responses between LIRT-BFR and traditional resistance training are scarce. Regarding HRV, both LIRT-BFR and low-intensity resistance training without BFR (LIRT) performed until concentric failure in a single exercise appear to elicit similar autonomic responses [31,32]. In contrast, RPE values during single-exercise protocols with four sets and a fixed number of repetitions tend to be higher in HIRT and LIRT-BFR compared with LIRT [24,32,33].

Although these studies provide important mechanistic information, they do not fully represent the cumulative demands of a complete resistance-training session. In practical settings, resistance training is commonly organized as a sequence of exercises involving upper- and lower-body muscle groups, repeated sets, short or moderate rest intervals, and progressive accumulation of fatigue. Under these conditions, the combined effects of metabolite accumulation, repeated cardiovascular adjustments, reduced recovery between sets, and concentric failure may produce perceptual and autonomic responses that differ from those observed after a single isolated exercise [3,15,34]. This distinction is particularly important when comparing HIRT, LIRT, and LIRT-BFR, because each protocol may impose a different balance between mechanical tension, metabolic stress, volume tolerance, and perceived exertion.

Therefore, the present exploratory crossover study aimed to compare the acute effects of HIRT, LIRT, and LIRT-BFR on HRV parameters, RPE, total workload, and number of repetitions during full resistance-training sessions composed of sequential upper- and lower-body exercises performed until concentric failure. We hypothesized that LIRT-BFR would elicit higher RPE than LIRT and values closer to HIRT, despite using the same relative load as LIRT. We also hypothesized that all protocols would reduce global HRV after exercise, but that between-protocol differences in HRV would be less evident than perceptual differences because of the exploratory sample size and the multifactorial nature of post-exercise autonomic recovery.

## 2. Materials and Methods

### 2.1. Participants

Thirteen young recreationally trained men were recruited for this study (age: 21.5 ± 1.6 years; height: 178.2 ± 8.0 cm; body mass: 75.7 ± 8.0 kg). The sample size was previously estimated using G*Power (v. 3.1.9.4, Heinrich-Heine-Universität Düsseldorf, Düsseldorf-Stadtbezirk, Germany) based on previous findings on the differences in the rate of perceived exertion (RPE) between high-load and low-load intensity training protocols (8.1 ± 0.6 vs. 6.2 ± 1.1, r^2^ = −0.46) [35], considering a confidence level (Z_1-alpha/2_ = 1.960) and a power of 95% (Z_1-beta_ = 1.645). The estimated sample size was 10 participants, which was increased by 30% (*N* = 13) to prevent statistical underpower due to participant dropout.

Eligibility criteria included: age between 18 and 30 years, regular participation in resistance training for at least six months, and no history of diseases such as diabetes, ischemia, arrhythmias, hypertension, or obesity (BMI ≥ 30 kg/m^2^). Additionally, individuals who had used medications such as beta-blockers or antihypertensives within the previous two months were excluded. Only volunteers who met all inclusion criteria were enrolled in the experimental protocol. All procedures, including potential risks, were clearly explained, and written informed consent was obtained from every participant prior to participation. The research was approved by the Ethics Committee of the Federal University of Alfenas (UNIFAL) (CAEE 76049423.6.0000.5142), performed in accordance with the ethical standards of the Helsinki Declaration.

### 2.2. Experimental Design

The study employed a crossover design in which each participant completed all experimental protocols. Data collection occurred over seven non-consecutive visits, with a minimum interval of 72 h between sessions. During the first four visits, participants underwent familiarization sessions with the exercises and performed one-repetition maximum (1RM) testing. The remaining three visits were dedicated to the experimental sessions: HIRT, LIRT, and LIRT-BFR, which were conducted at the same time of day for each participant to minimize physiological variability and circadian-related effects.

### 2.3. One-Repetition Maximum Testing

The 1RM assessment was conducted following the protocol proposed by Macedo et al. [35] to determine the maximal load for each exercise used in the experimental training sessions. The test began with a general warm-up, followed by specific warm-up sets with progressively increasing loads until reaching the participant’s maximal concentric capacity. Participants were allowed three to five attempts per exercise, with no more than two repetitions per set. Rest intervals were set at 90 s between warm-up sets and 180 s between maximal attempts. During each testing session, two exercises were assessed (one for the upper body and one for the lower body) with 72 h of recovery between testing visits.

### 2.4. Training Sessions

The training sessions followed a full-body model based on the protocol proposed by Lopes et al. [36] and included six exercises targeting both upper- and lower-limb muscle groups. The exercise sequence was as follows: machine bench press, lat pulldown, lateral raise, standing leg curl, horizontal leg press, and hack machine calf raise. The organization of loads, number of sets, and rest intervals was defined according to contemporary recommendations for muscle hypertrophy in traditional resistance training and resistance training combined with BFR [1,2]. Each participant completed three distinct training protocols, which were performed in a non-randomized and non-counterbalanced order as follows: HI, LI, and LIBFR [37], with a 72-h washout period respected between sessions.

In the HIRT protocol, exercises were performed at 70% of 1RM for four sets, with 90 s of rest between sets and 120 s between exercises. In the LIRT protocol, participants used 30% of 1RM, also for four sets, with a shortened rest interval of 30 s between sets and 120 s between exercises. The LIRT-BFR protocol was performed with 30% of 1RM under blood flow restriction maintaining the same number of sets and rest intervals as LIRT.

**Figure 1:**
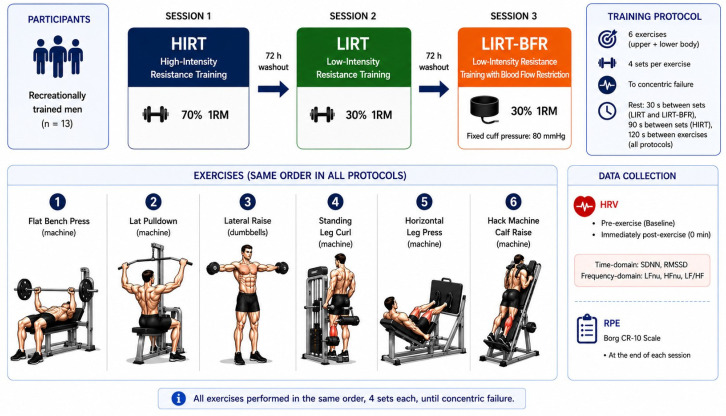
Schematic overview of the experimental design, resistance-training protocols (HIRT, LIRT, and LIRT-BFR), exercise sequence, and outcome assessments. This Figure was created using the image-generation tool integrated into ChatGPT (OpenAI, version GPT—5.5, 2026) and subsequently refined by the authors for layout and labeling.

All training protocols were performed in repetition-to-failure mode, with failure defined as the inability to complete the concentric phase within the standardized movement cadence (1 s concentric and 2 s eccentric), as recommended for resistance training execution [38]. A schematic overview of the experimental design, training protocols, and outcome assessments is presented in Figure 1.

### 2.5. Blood Flow Restriction

The BFR was applied using cuffs designed specifically for each body segment. For upper-limb exercises, a cuff measuring 80 cm × 7 cm (Scientific-arm-WCS, Cardiomed^®^, Curitiba, Brazil) was used, whereas for lower-limb exercises, cuffs measuring 84 cm × 12.5 cm (Scientific-leg-WCS, Cardiomed^®^, Curitiba, Brazil) were employed. During upper-limb exercises, cuffs were positioned on the proximal region of the arm, just below the deltoid insertion, encompassing the upper portion of the biceps brachii. For lower-limb exercises, cuffs were placed on the proximal thigh, immediately below the inguinal ligament, covering the upper quadriceps region [27,35]. In the LIRT-BFR protocol, cuff pressure was fixed at 80 mmHg for all participants [39,40]. The cuffs remained inflated continuously during exercise execution and rest intervals between sets and were deflated only during transitions between different exercises [35].

Although previous studies recommended to individualize pressure based on a percentage of arterial occlusion pressure (AOP) (40–80% of AOP), this procedure could not be applied during the LIRT-BFR protocol. Measuring AOP requires Doppler ultrasound and a trained evaluator, which could introduce additional variability [41]. Furthermore, the exercises in the present protocol were performed in different body positions (seated, standing, and supine), which substantially alters AOP values [42,43]. Considering these practical limitations, a fixed pressure standardization was selected.

### 2.6. Rating of Perceived Exertion (RPE)

RPE was assessed using the Borg CR10 scale (0–10). After completing the four sets of each exercise, participants verbally reported the value corresponding to their perceived exertion. The session-level RPE was obtained by averaging the RPE values assigned to each exercise, following the methodology described by Macedo et al. [35].

### 2.7. Heart Rate Variability Recording

Participants were instructed to avoid physical exercise and caffeine consumption for 24 h before data collection, following the recommendations of Ernst [15]. Before measurements, volunteers remained seated at rest for 15 min. For baseline assessment, R-R intervals were recorded for 5 min in a seated position while maintaining a controlled respiratory rate between 9 and 22 breaths per minute. After completion of the experimental training sessions, data were recorded again for 5 min, in the same seated position and using the same equipment [14].

Recordings were obtained using a Polar RS800CX heart rate monitor (Polar Electro, Kempele, Finland) and analyzed with Polar ProTrainer 5 software (version 4.0, Kempele, Finland). A stationary 5-min segment was selected after linear interpolation of adjacent intervals and processed using Kubios HRV software (version 2.1, Biosignal Analysis and Medical Imaging Group, Kuopio, Finland). Time-domain indices included SDNN and RMSSD as markers of parasympathetic modulation. Frequency-domain variables included HFnu (0.15–0.40 Hz), indicative of vagal activity; LFnu (0.04–0.15 Hz), associated with sympathetic predominance; and the LF/HF ratio, representing cardiac sympathovagal balance.

The interpretation of HRV indices, particularly LFnu and the LF/HF ratio, remains a subject of ongoing debate. Although traditionally linked to sympathetic modulation and sympathovagal balance, recent evidence demonstrates that such interpretations are overly simplistic. The LF component reflects mixed autonomic influences—including vagal participation, baroreflex activity, and respiratory factors—rather than serving as an exclusive marker of cardiac sympathetic activity [44,45,46]. Similarly, the LF/HF ratio must be interpreted with caution due to confounding physiological and methodological factors, such as respiratory rate [14,47]. Nonetheless, when analyzed alongside time-domain parameters (e.g., SDNN and RMSSD) and within the specific experimental context, these indices remain valuable complementary tools for autonomic assessment.

### 2.8. Statistical Analysis

All data are presented as mean ± standard deviation (SD). Normality was assessed using the Shapiro–Wilk test, and homogeneity of variances was examined using Levene’s test for one-way ANOVA and Mauchly’s test for two-way ANOVA. A one-way repeated-measures analysis of variance (ANOVA) was used to compare the HIRT, LIRT, and LIRT-BFR protocols for total workload, total number of repetitions, and mean RPE. A two-way repeated-measures ANOVA was performed to analyze the interaction between protocol pre- and post-exercise for HRV parameters (SDNN, RMSSD, LFnu, HFnu and LF/HF). When significant main or interaction effects were detected, Bonferroni-adjusted post hoc tests were applied (*α* < 0.05). Effect sizes for one-way and two-way repeated-measures ANOVA were calculated using partial eta squared (η^2^_p_), with thresholds interpreted as follows: ≥0.01 indicates a small effect, ≥0.06 a medium effect, and ≥0.14 a large effect. These values represent the rounded mathematical conversions of Cohen’s f thresholds (0.10, 0.25, and 0.40, respectively) into partial eta squared (η^2^_p_) [48]. The power for all comparisons was determined using G*Power software (v. 3.1.9.4) with a 95% confidence level (α = 0.05) and η^2^p values reported for all analyses. The power for all comparisons was determined using G*Power software (v. 3.1.9.4) with a 95% confidence level (α = 0.05) and η^2^p values reported for all analyses. All analyses were performed using SPSS software version 26.0 (IBM Corp., Armonk, NY, USA).

## 3. Results

**Figure 2:**
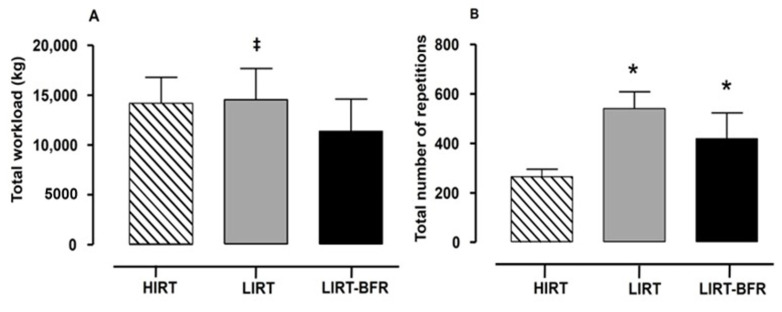
Total workload (**A**) and total number of repetitions (**B**) obtained during the resistance training sessions in the high-intensity (HIRT), low-intensity (LIRT), and low-intensity with blood flow restriction (LIRT-BFR) protocols. ‡ Significant difference (*p* < 0.05) compared with LIRT-BFR. * Significant difference (*p* < 0.05) compared with HIRT.

Figure 2 presents the total workload values for the HIRT, LIRT, and LIRT-BFR training sessions (F_[2,36]_ = 4.358; *p* = 0.020; η^2^_p_ = 0.195 [large]; power = 0.72; Panel A), and the total number of repetitions performed during each training session (F_[2,36]_ = 44.647; *p* = 0.001; η^2^_p_ = 0.713 [large]; power = 1.00; Panel B). As shown, total workload was significantly higher in the LIRT protocol compared with LIRT-BFR (*p* < 0.05), with no difference relative to HIRT (*p* > 0.05) (Panel A).

**Figure 3:**
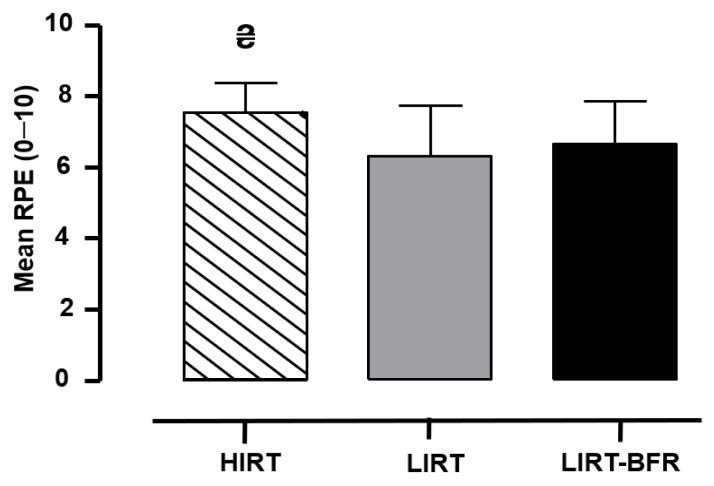
Rating of perceived exertion (RPE) values during the resistance training sessions in the high-intensity (HIRT), low-intensity (LIRT), and low-intensity with blood flow restriction (LIRT-BFR) protocols. ₴ Significant difference compared with LIRT (*p* < 0.05).

Figure 3 presents the mean RPE values for the three protocols. LIRT showed the lowest mean RPE (6.5) compared with HIRT (7.8) and LIRT-BFR (7.1), with significant differences (F_[2,36]_ = 3.360; *p* = 0.036; η^2^_p_ = 0.169 [large]; power = 0.64) among protocols (*p* < 0.05).

HRV results are presented in Table 1. Time-domain parameters (SDNN and RMSSD) and frequency-domain components (LFnu, HFnu, and LF/HF) were analyzed. SDNN showed a significant reduction from pre- to post-exercise (F__[2,35]__ = 22.770; *p*= 0.001; η^2^_p_ = 0.394 [large]) in all protocols—HIRT (*p* < 0.001), LIRT (*p* < 0.001), and LIRT-BFR (*p* < 0.001)—with no differences among protocols (F__[2,35]__ = 2.110; *p*= 0.136; η^2^_p_ = 0.108 [medium]; power = 0.40). For RMSSD (F_[2,35]_ = 0.080; *p* = 0.924; η^2^_p_ = 0.005 [trivial]), LFnu (F_[2,35]_ = 0.748; *p* = 0.481; η^2^_p_ = 0.041 [small]), HFnu (F_[2,35]_ = 1.262; *p* = 0.296; η^2^_p_ = 0.067 [medium]), and LF/HF (F_[2,35]_ = 2.407; *p* = 0.105; η^2^_p_ = 0.121 [medium]), no pre–post differences were identified in any protocol (*p* > 0.05).

Similarly, no differences among protocols were observed for RMSSD (F_[2,35]_ = 1.722; *p* = 0.194; η^2^_p_ = 0.090 [medium]), LFnu (F_[2,35]_ = 0.731; *p* = 0.489; η^2^_p_ = 0.040 [small]), HFnu (F_[2,35]_ = 0.450; *p* = 0.641; η^2^_p_ = 0.025 [small]), or LF/HF (F_[2,35]_ = 0.070; *p* = 0.932; η^2^_p_ = 0.004 [trivial]), in either pre- or post-exercise measurements (*p* > 0.05).

## 4. Discussion

The present study demonstrated that the LIRT session elicited lower RPE values compared with HIRT and LIRT-BFR. This finding suggests that, under the present experimental conditions, low-load resistance exercise performed without vascular restriction was perceived as less demanding, even when sets were performed to concentric failure.

In contrast, HRV responses showed a post-exercise reduction exclusively in SDNN. This finding should be interpreted as a decrease in overall HRV, reflecting the expected shift toward elevated sympathetic stress and attenuated vagal modulation recovery rather than as evidence of decreased sympathetic tone. No statistically significant between-protocol differences were detected for the remaining HRV indices, including RMSSD, LFnu, HFnu, and LF/HF. Therefore, the present findings should not be interpreted as proof of equivalent autonomic stress among protocols, but rather as an absence of detectable differences within the limits of the sample size, measurement procedures, and acute post-exercise assessment used in this exploratory crossover study [3,4,34,49,50].

Additionally, total workload was higher in LIRT compared with LIRT-BFR, with no difference relative to HIRT, while the total number of repetitions was lower in HIRT. Across the sets, total workload and repetitions progressively declined in all three protocols: HIRT, LIRT, and LIRT-BFR. Taken together, these findings indicate that resistance-training protocols with different combinations of external load, vascular restriction, and repetition capacity may generate distinct mechanical and perceptual profiles, even when performed within the same full-body session structure.

Previous studies examining total workload in high-intensity resistance training and BFR training using a single exercise, without reaching concentric failure, have reported higher workload values in high-intensity protocols [35]. Similar patterns have been observed in protocols conducted to concentric failure, where the high-intensity protocol produced greater total workload and number of repetitions compared with BFR protocols [50]. However, the present findings add an important nuance to this interpretation. Although HIRT involved a higher relative load, the lower number of repetitions performed in this condition attenuated differences in total workload when compared with LIRT. Conversely, LIRT allowed a greater number of repetitions because of the lower relative load, which may explain why total workload was maintained or even exceeded that observed in LIRT-BFR. These findings are consistent with the principle that total workload in resistance exercise is determined not only by the external load, but also by the number of repetitions completed, the number of sets, rest intervals, and the proximity to concentric failure [17,25,51]. In the LIRT-BFR condition, the lower total workload compared with LIRT may reflect the additional fatigue and discomfort imposed by vascular restriction, which can reduce repetition tolerance despite the same relative load. Thus, rather than indicating that one protocol is globally more demanding than another, the present results suggest that HIRT, LIRT, and LIRT-BFR impose different combinations of mechanical tension, repetition volume, metabolic stress, and perceptual strain.

RPE values were lower in the low-intensity protocol compared with the high-intensity and BFR protocols. This finding aligns with the literature, which consistently shows that perceived exertion increases proportionally with relative load, with higher-load exercises being accompanied by greater perceived effort [11,12,13]. In HIRT, the elevated RPE is plausibly explained by the higher external load, greater central motor command, higher mechanical tension, and progressive recruitment of high-threshold motor units as fatigue accumulates across sets. In HIRT, the elevated RPE is plausibly explained by the higher external load, greater central motor command, higher mechanical tension, and progressive recruitment of high-threshold motor units as fatigue accumulates across sets. BFR protocols, even when performed with low loads, also tend to produce high RPE values due to metabolite accumulation, local hypoxia, and increased recruitment of fast-twitch motor units—factors that enhance discomfort [16,23].

The perceptual response to LIRT-BFR may also be influenced by the activation of group III and IV muscle afferents, which respond to mechanical distortion, ischemia, and metabolite accumulation, contributing to the exercise pressor reflex and to sensations of effort, pain, and local discomfort [21]. Therefore, the similar or elevated RPE observed in LIRT-BFR compared with HIRT should not be interpreted as a contradiction of its low-load nature. Instead, it reinforces that external load and internal perceptual strain are not interchangeable constructs. A protocol may use low mechanical load while still imposing substantial local metabolic and perceptual stress.

This distinction is clinically and practically relevant. In rehabilitation or return-to-training contexts, LIRT-BFR is often proposed as an alternative when high mechanical loads are not tolerated or are temporarily contraindicated [16]. However, the present findings suggest that the reduced external load of BFR should not automatically be equated with lower perceived difficulty. For patients with low discomfort tolerance, cardiovascular vulnerability, or limited familiarization with occlusion training, the perceptual burden of BFR may influence adherence and session quality. Conversely, for trained individuals or athletes, LIRT-BFR may be useful when the goal is to maintain a high internal stimulus while reducing joint and connective tissue loading. This may be particularly relevant during deloading phases, injury recovery, or periods in which high-load resistance training needs to be temporarily reduced. Recent meta-analytic evidence suggests that BFR combined with low-load resistance training can improve strength and muscle size, although high-load training may remain superior for maximal strength development in some contexts, and methodological factors such as cuff width, pressure prescription, training status, and proximity to concentric failure can influence outcomes [16,18,19].

HRV results showed a significant reduction in the standard deviation of NN intervals (SDNN) from pre- to post-training in all protocols, with no differences among them. SDNN represents the global variability of heart rate and is influenced by multiple physiological inputs, including parasympathetic modulation, sympathetic modulation, respiratory influences, baroreflex activity, and the overall recovery state [4]. Accordingly, the post-exercise reduction in SDNN observed in the present study should be described conservatively as an acute reduction in global HRV following resistance exercise. This response is compatible with transient cardiovascular perturbation after exercise, but it does not allow precise attribution to a single autonomic branch. Previous evidence indicates that acute resistance exercise can reduce HRV after exercise, and that the magnitude of this response may depend on exercise volume, intensity, rest intervals, muscle mass involved, and recovery duration [3,45]. Because all three protocols in the present study involved six exercises, four sets per exercise, and sets performed to concentric failure, the cumulative full-session demand may have been sufficient to reduce global HRV regardless of the specific loading strategy.

In contrast, the time-domain parameter RMSSD and the frequency-domain components LFnu, HFnu, and LF/HF ratio did not show significant pre–post changes or differences among protocols. RMSSD is commonly considered more closely related to short-term vagal modulation than SDNN, whereas frequency-domain indices are more sensitive to methodological factors such as respiratory pattern, recording duration, posture, and signal processing procedures [14,15]. Therefore, the absence of significant changes in RMSSD, LFnu, HFnu, and LF/HF should be interpreted cautiously. It may indicate that these indices were less responsive than SDNN in the present protocol, but it may also reflect limited statistical power, interindividual variability, or insufficient sensitivity of the measurement window to detect protocol-specific differences. Similar concerns apply to LF/HF, which should not be interpreted as a direct measure of sympathovagal balance because this ratio is influenced by complex physiological and mathematical interactions and does not provide a valid isolated estimate of sympathetic or parasympathetic activity [45]. Thus, the present HRV findings support the conclusion that all protocols reduced global HRV acutely, but they do not support strong claims regarding equivalent autonomic stress or specific sympathetic–parasympathetic mechanisms across HIRT, LIRT, and LIRT-BFR.

Another important aspect is that perceptual and autonomic markers did not follow identical patterns. LIRT produced lower RPE than HIRT and LIRT-BFR, whereas HRV indices did not differ between protocols. This dissociation suggests that RPE and HRV capture different dimensions of the acute training response. RPE integrates local muscular discomfort, effort perception, central command, respiratory strain, and psychological tolerance, whereas HRV reflects cardiac autonomic modulation during recovery. Therefore, the combined use of RPE and HRV may provide complementary information for monitoring resistance-training sessions, particularly when comparing protocols with different mechanical and metabolic characteristics [5,7,9]. In practical terms, coaches and clinicians should not rely exclusively on HRV to infer perceived difficulty, nor should they assume that a lower-load session necessarily produces lower internal stress when BFR is applied.

RPE values were lower in the low-intensity protocol compared with the high-intensity and BFR protocols. This finding aligns with the literature, which consistently shows that perceived exertion increases proportionally with relative load, with higher-load exercises being accompanied by greater perceived effort [21]. BFR protocols, even when performed with low loads, also tend to produce high RPE values due to metabolite accumulation, local hypoxia, and increased recruitment of fast-twitch motor units—factors that enhance discomfort [51]. Therefore, the lower RPE observed in the low-intensity protocol reflects a combination of moderate intensity and the absence of vascular occlusion, conditions that reduce perceptual stress.

HRV results showed a significant reduction in the standard deviation of NN intervals (SDNN) from pre- to post-training in all protocols, with no differences among them. SDNN represents the global variability of heart rate and is influenced by both sympathetic and parasympathetic modulation. Its reduction after exercise suggests a transient increase in sympathetic activity and decreased vagal modulation, a typical response following intense physical effort [23,52,53].

In contrast, the time-domain parameter RMSSD and the frequency-domain components (LFnu, HFnu, and LF/HF ratio) did not show significant pre–post changes or differences among protocols. These findings suggest that the autonomic stress induced by the training sessions was not sufficiently intense or prolonged to affect these indices. Similar results were reported by Kingsley et al. [6], who observed that single resistance training sessions, even when performed to failure, reduce global HRV but do not significantly alter indices related to vagal modulation.

### 4.1. Limitations

The present study has limitations that should temper interpretation. First, the current sample size limits generalizability and increases the risk of type II error, particularly for HRV variables with high interindividual variability. Second, regarding the blood flow restriction protocol, a technical limitation involves the use of a fixed pressure of 80 mmHg used in this study, which does not individualize relative pressure among participants. Although this absolute pressure does not account for individual occlusion thresholds, it directly aligns with the absolute pressure ranges (50–200 mmHg) previously validated in the literature [54,55]. Since BFR benefits have been demonstrated at pressures as low as 50 mmHg, our standardized approach ensures both efficacy and practical application across different exercise positions without the methodological constraints of AOP measurement. Another limitation was the absence of experimental groups without concentric failure, which prevented the isolation of the specific effect of concentric failure on autonomic responses. Additionally, although breathing rate was controlled within a predefined range, respiratory variation may still have influenced HRV indices, particularly frequency-domain variables.

Respiratory frequency is a well-established modulating factor of HRV, especially for frequency-domain indices (e.g., HFnu, LFnu, and LF/HF ratio), and spontaneous variations in ventilation can alter these parameters independently of true cardiac autonomic shifts [14,56]. While establishing a stringent respiratory control (such as guided breathing) is highly recommended to reduce confounding variables [15], we attempted to mitigate this by ensuring an identical, quiet, and stable environment during all collections. Nonetheless, the wide spontaneous respiratory range should be considered when interpreting the present autonomic outcomes. Fourth, the inclusion of only young recreationally trained men limits extrapolation to women, older adults, clinical populations, and athletes with different training backgrounds. Finally, the acute design prevents conclusions regarding chronic adaptations in strength, hypertrophy, vascular function, recovery, or autonomic modulation. Future studies should use larger and more diverse samples, individualized BFR pressures, stricter respiratory control, additional physiological markers such as blood lactate, blood pressure, muscle oxygenation, and neuromuscular fatigue, and longitudinal designs to determine whether acute perceptual and autonomic responses predict chronic training adaptations.

Furthermore, some limitations regarding the interpretation of frequency-domain HRV indices must be acknowledged. Although LFnu and the LF/HF ratio have traditionally been used as markers of sympathetic predominance and sympathovagal balance, current consensus urges caution. Recent evidence suggests that the LF component reflects a complex interaction among sympathetic and parasympathetic mechanisms, baroreflex activity, and respiratory influences, rather than serving as an isolated marker of cardiac sympathetic drive [40,41]. Similarly, the physiological validity of the LF/HF ratio as a quantitative index of autonomic balance has been challenged, especially under experimental conditions involving acute physical exercise and post-exercise recovery [12,56]. Therefore, the lack of significant differences in spectral HRV indices among the evaluated protocols does not necessarily imply a complete equivalence in the autonomic stress induced by the training sessions. Consequently, these outcomes should be interpreted integrally alongside the other physiological and perceptual responses observed.

Despite these limitations, the present study contributes to the literature by comparing HIRT, LIRT, and LIRT-BFR within the same experimental framework using a full-body resistance-training session performed to concentric failure. This design more closely resembles applied training practice than single-exercise models and allows a more ecologically valid comparison of perceptual, mechanical, and autonomic responses. From an applied perspective, LIRT may be preferable when the goal is to reduce perceived exertion while maintaining repetition volume; HIRT remains relevant when high mechanical tension is desired; and LIRT-BFR may be useful when high external loads are undesirable but a substantial internal stimulus is still intended. However, because LIRT-BFR may increase perceived effort despite low external load, practitioners should individualize its use according to training status, clinical condition, discomfort tolerance, and recovery objectives.

### 4.2. Strength and Future Research Lines

A major strength of this study is the direct comparison of HIRT, LIRT, and LIRT-BFR within the same experimental framework. This design allows for a nuanced understanding of how different loading strategies influence perceptual, autonomic, and mechanical responses. By integrating the RPE, HRV, and total workload and repetitions performed by the athletes, the study provides a robust and holistic evaluation of internal and external load across protocols. Additionally, the crossover experimental design reduced the interindividual variability and increased the sensitivity to detect true differences between training conditions, strengthening the reliability of the findings.

Future studies should carry out longitudinal research to determine whether the acute differences observed in RPE, workload, and HRV translate into divergent long-term adaptations in strength, hypertrophy, fatigue management, or autonomic function. In order to generalize the results and increase the study samples, future research could analyze populations with disabilities, individuals with limited load tolerance or metabolic disorders. Also, more physiological and neuromuscular markers could be included in the study, such as blood lactate, muscle oxygenation or biochemical markers of stress and fatigue.

It is worth noting that while the sample size was calculated a priori based on RPE scores, the observed power and effect sizes for the HRV parameters were reported herein. Although post hoc power interpretation should be approached with caution regarding current hypotheses, providing these statistical metrics is intended to serve as a robust empirical reference for future counterbalanced or crossover trials aiming to perform precise a priori sample size estimations for cardiac autonomic variables.

### 4.3. Practical Applications

From a practical perspective, the present findings may help clinicians, coaches, and practitioners tailor resistance-training prescription according to mechanical, perceptual, and recovery demands. In rehabilitation settings, older adults, or individuals recovering from injury, LIRT-BFR may represent a valuable strategy to induce substantial neuromuscular and metabolic stimulation while minimizing external load and joint stress, thereby favoring strength and hypertrophy adaptations without excessive mechanical overload on healing tissues. For athletic populations, HIRT and LIRT-BFR may be strategically alternated according to the objectives of the training plan. While HIRT remains important for maximizing mechanical tension and strength development, LIRT-BFR may provide an alternative to maintain metabolic stimulus and muscle mass during tapering periods, congested competitive schedules, travel, or phases of reduced load tolerance. Additionally, because no clear between-protocol differences were detected in short-term autonomic recovery within the conditions of the present study, practitioners may select training strategies according to perceptual and mechanical fatigue constraints. For example, LIRT may be preferable during periods of elevated psychological fatigue due to its lower perceived exertion, whereas LIRT-BFR may be useful when the goal is to maintain a high internal metabolic stimulus while reducing total mechanical workload and joint stress compared with traditional high-intensity resistance training.

## 5. Conclusions

The present findings indicate that full-body resistance training sessions composed of upper- and lower-limb exercises performed until concentric failure did not demonstrate statistically detectable between-protocol differences in post-exercise HRV responses among HIRT, LIRT, and LIRT-BFR under the conditions of the present study. In contrast, perceptual and performance-related responses differed across protocols, as LIRT elicited lower RPE values, whereas LIRT-BFR was associated with reduced total workload despite a similar repetition performance. These findings suggest that different resistance training strategies may produce distinct combinations of mechanical, perceptual, and metabolic demands, even when acute autonomic recovery responses appear broadly comparable within the limits of the present experimental design.

Importantly, these findings should be interpreted cautiously due to the exploratory nature of the study, the relatively small sample size and limited statistical power for some HRV outcomes, and the use of a fixed rather than individualized BFR pressure prescription. Nevertheless, the study provides ecologically relevant evidence by comparing traditional and blood flow restriction resistance training during whole-body sessions performed to concentric failure, while simultaneously examining perceptual, performance, and short-term cardiovascular autonomic responses. From an applied perspective, the results support the strategic use of HIRT, LIRT, and LIRT-BFR according to mechanical load tolerance, perceptual fatigue, and exercise prescription goals in trained young adults. Future studies with larger and more diverse samples, individualized BFR pressures, and longitudinal designs are warranted to clarify the chronic implications of these acute responses.

## Figures and Tables

**Table 1:** Heart rate variability (HRV) parameters in the HIRT, LIRT, and LIRT-BFR protocols.

	HIRT	LIRT	LIRT-BFR	*p*
SDNN				
Pre	51.5 ± 21.7	62.3 ± 38.4	71.1 ± 34.6	<0.001 ^₶^
Post	27.1 ± 21.8	30.8 ± 24.0	35.0 ± 32.6	
RMSSD				
Pre	42.1 ± 20.3	55.5 ± 45.2	62.3 ± 37.9	>0.05
Post	25.6 ± 31.4	39.0 ± 29.3	50.4 ± 34.9	
LFnu				
Pre	68.0 ± 20.8	67.8 ± 19.4	64.7 ± 21.9	>0.05
Post	73.6 ± 24.4	64.3 ± 26.2	61.5 ± 23.6	
HFnu				
Pre	30.2 ± 17.8	26.9 ± 12.5	30.9 ± 18.0	>0.05
Post	22.4 ± 19.4	32.0 ± 23.8	34.7 ± 21.1	
LF/HF				
Pre	3.5 ± 1.9	3.1 ± 1.8	7.4 ± 12.9	>0.05
Post	6.7 ± 5.2	6.0 ± 6.6	3.9 ± 4.0	

Note: Values are expressed as mean ± standard deviation. RRi = RR interval; SDNN = standard deviation of NN intervals; RMSSD = root mean square of successive differences; LFnu = normalized low-frequency component; HFnu = normalized high-frequency component; LF/HF = low-frequency to high-frequency ratio. ₶ Significant difference compared with the pre-exercise moment (*p* < 0.05). For all HRV indices, the observed statistical power for pre-to-post comparisons reached 100%, whereas for between-protocol comparisons, it ranged from 6% to 40%.

## Data Availability

The data that support the findings of this study are available from correspondence author (dalton.pessoa-filho@unesp.br) upon reasonable request.

## Abbreviations

The following abbreviations are used in this manuscript:

BFR	Blood Flow Restriction
RT	Resistance Training
HIRT	High-Intensity Resistance Training
LIRT	Low-Intensity Resistance Training
LIRT-BFR	Low-Intensity Resistance Training with Blood Flow Restriction
RPE	Rate of Perceived Exertion
[La-]	Blood Lactate Accumulation
SBP	Systolic Blood Pressure
AOP	Arterial Occlusion Pressure
1RM	One repetition to maximum
HRV	Heart rate variability
RRi	RR interval
SDNN	Standard deviation of NN intervals
RMSSD	Root mean square of successive differences
LFnu	Normalized low-frequency component
HFnu	Normalized high-frequency component
LF/HF	Low-frequency to high-frequency ratio
